# The History of the Development of the Ovum of the Dog, with Fifteen Lithographic Plates

**Published:** 1847-07-01

**Authors:** 


### MEDICO-CHIRURGICAL REVIEW.

JULY, 1847.

I. ENTWICKELUNGSGESCHICHTE DES HUNDE-EIES. Von *Th. L. W. Bischoff.*

The History of the Development of the Ovum of the Dog, with Fifteen Lithographic Plates. By Dr. *T. L. W. Bischoff*, Professor of Medicine, &c. in the University of Giessen. Brunswick, 1845.

II. ZUSÄTZE ZUR LEHRE VOM BAUE UND VON DEN VERRICHTUNGEN DER GESCHLECHTSORGANE. Von *Ernst Heinrich Weber* in Leipzig. Müller's Archiv. für Anatomie, 1846.

Contributions to illustrate the Structure and Functions of the Sexual Organs. By Professor *E. H. Weber.*

III. UEBER DIE GLANDULÆ UTRICULARES DES UTERUS DES MENSCHEN UND IHREN ANTHEIL AN DER BILDUNG DER DECIDUA. Von *Th. Ludw. W. Bischoff.* Müller's Archiv. 1846.

On the Glandulæ Utriculares of the Human Uterus, and on the Share they take in the Formation of the Decidua. By Professor *Bischoff.*

IV. DIE ENTWICKELUNG DES MENSCHEN UND DES HÜHNCHENS IM EIE, &c. Von Dr. *M. P. Erdl.* Leipzig, 1845-46.

On the Development of the Human Embryo, and of the Chick in the Ovum. By Professor *M. P. Erdl.*

THE anatomy of the present day, when contrasted with that taught a quarter of a century ago, is eminently distinguished by its scientific, or, to speak more accurately, by its philosophic character; a change which has been brought about more especially by the increased attention that has of late years been devoted to those branches of organization upon which the whole subject, as a science, must eventually rest—structural anatomy, embryology, and comparative anatomy. Some have indeed questioned the advantage of the change as to any practical benefits that have been attained; but whatever regrets of this kind may in certain quarters have been expressed, they can now have no result—the new direction impressed upon the study of anatomy having become what our nearest neighbours term "*un fait accompli.*"

Of the three divisions of anatomical science just named, Embryology may, in some and not unimportant points, be regarded as the most essential to the elucidation of the general laws of organization.

To those indeed whose attention has been mainly restricted to human anatomy, the history of the development of the ovum has, for the most part, been regarded only so far as of importance, as it tends to explain the formation of the various membranes of the fœtus, and the relations existing between the latter and the uterus. But this inquiry has a much wider signification; for, inasmuch as it reveals the typical forms of the several classes of organs, and their subsequent modifications and metamorphoses, such an investigation assumes a much more imposing character, and by leading the observer to discriminate between the essential and the incidental, enables him to determine the fundamental principles of animal formation. It must clearly have been the perception of this truth, joined to the general interest attaching to the production of the new being, which has excited the most distinguished physiologists of all countries, among whom it will suffice to name Harvey, Malpighi, and Hunter, to scrutinise with such persevering zeal the process of generation.

From what has been now stated, it is apparent that the study of Embryology divides itself into two branches:—one, and by far the most important and abstruse department, relates to the general laws of formation, to the essential construction of animal bodies and of their individual organs, and thus becomes, with its inseparable ally, comparative anatomy, the basis of all philosophic zoology; the second division has reference not to the development of the new being, but to the numerous and complex phenomena constituting the conditions upon which that development depends, including the various nutritive and defensive provisions by which the embryo is nourished and protected. In the present article, we shall, for the most part, confine ourselves to this latter branch of the inquiry, as being the more interesting of the two to the medical practitioner; it will, however, be proper to notice some points connected with the more comprehensive division of the subject.

It cannot have escaped the attention even of those who have considered this inquiry only in a cursory manner, that, owing to obvious causes, by far the larger portion of the information we now possess touching the constitution and development of the ovum, has been gained rather by observations made on the reproductive process of the lower animals than by any direct results that have been attained from the study of human embryology. The egg of the common fowl has been more particularly selected from the earliest periods, not less on account of its size than of the facility of making repeated examinations at successive and known epochs in the progress of its evolution. Some of the most successful inquirers of the present day, among whom we may name the justly celebrated Baer, also W. Jones, Coste, Valentin, M. Barry, and Bischoff, have, however, followed in the steps of the admirable De Graaf, of whom a distinguished physiologist justly remarks, that he "has made a series of researches upon the first consequences of conception, which have been equalled by few of his successors and surpassed by none."* They have thus obtained many accurate details from the patient examination of the minute mammiferous ovum; nay, even some of the best examples of the earlier changes in the egg consequent upon impregnation, have been derived from the investigation of other diminutive ova, as those of the acalephæ by Siebold, of the entozoa by Bagge, and of insects by Kölliker.

So large a part of our present knowledge thus resting on comparative embryology, and as in the human being all accurate investigation is opposed by so many obstacles, some of which are insuperable, it becomes a point of the first importance to know how far the deductions drawn from the study of the lower animals, can be applied to man. The only mode in which this prime question can be solved, is by considering, in the first place, what are the component parts of the egg in the different classes of the animal kingdom; and, in the second place, what are the changes consequent upon the fertilization of the ovum. In the prosecution of these inquiries, it will be necessary to enter into details at the hazard of stating facts that are perhaps familiar to some of our readers, but which, notwithstanding their important bearing upon the whole question, have not, in our estimation, received the attention they demand. These preliminary inquiries will, at the same time, form the most natural introduction to the important work of Professor Bischoff, to which we design more particularly to direct the attention of our readers.

Touching the essential parts of the ovum, there is no doubt of their universal existence in all animals. By these parts are meant the germinal vesicle together with its macula, and the yelk with its membrane; these are met with equally in the vertebrata and invertebrata, in the smallest as in the largest eggs. The constant presence of these parts points to their supreme importance; and the progress of modern science, although it leaves the question of the first origin of the embryo in its original obscurity, still enables us to interpret the respective shares which the parts just named take in the subsequent development of the germ. The germinal vesicle, first discovered in 1825 by Purkinje in the cicatricula of the hen's egg, and subsequently and independently by Wharton Jones, Coste, and Valentin, in the ovum of mammals, is, with its contained germinal spot (discovered by Rudolph Wagner), that part of the egg which subsequently, by a process not even yet thoroughly known, becomes transformed into the rudiments of the germ. As to the yelk or vitellus, it takes no *direct* part either in the primitive formation or in the subsequent growth of the embryo; but it is essential inasmuch as it furnishes, either wholly or for a time, the nutritious matter required for the support and development of the new being.

In the case of all oviparous animals, this relation of the vitellus to the nourishment of the embryo, is immediately apparent; and it will subsequently appear that many important acts of development, which take place in mammals as the ovum traverses the Fallopian tube, and even for a part of the time, whilst it is still unattached in the uterus, are effected at the

* Handbuch der Entwickelungsgeschichte, von Dr. G. Valentin, p. 32.

expense of the yelk, which body furnishes the materials for the formation of the earliest and most fundamental parts of the germinal structures. It is remarkable that when Baer, repeating more carefully and skilfully the observations of Prevost and Dumas, first discovered the minute ovum of mammalia, he thought that this object corresponded to the germinal or Purkinjean vesicle of other animals, an error into which he could not have fallen, if the true office of the yelk, namely that of nourishing the germ in the first stages of its existence, had, at that time, been comprehended; so important is it, if it be wished to obtain a clear view of the whole process, scrupulously to investigate the very earliest changes that occur.

The constituent parts just noticed are then met with in the ovum of all animals, whilst it is still contained in the ovarium; and, as far as the germinal vesicle and its spot are concerned, there is tolerable uniformity of character throughout, the principal difference in the several classes depending on the relative size of the vitellus. Those who wish to satisfy themselves of this identity, may compare the small, greyish, and incipient eggs seen in the ovarium of birds with those of mammalia. Up to this point all is certain and undisputed; but in most animals, and especially in the ovipara, the ovum, after it has quitted the ovarium, receives, in its transit through the oviduct, and before it enters the homologous part to the uterus, certain additions, which are most apparent in birds. In this class it is well known that besides the chalazæ, which are evidently a mechanism peculiar to incubating animals, the upper part of the oviduct 'secretes the albumen or white, and the lower part called isthmus placed just above the uterus, the membrana testæ or chorion. We shall not pause to show that in many other oviparous classes both of these superadditions are made to the ovarian ovum, but proceed to consider what takes place in the case of mammals. Professor Owen, in his account of certain uterine ova which he examined in some pregnant ornithorhynchi, varying in diameter from $2\frac{1}{2}$ to $3\frac{1}{2}$ lines, states that there were two distinct membranes; the outer one is called the "chorion," and resembles the cortical membrane of the ovum of the salamander, except that it is of a more delicate texture; the inner membrane is regarded by Mr. Owen to be the membrana vitelli. Lying immediately beneath the latter, there was a thicker granular membrane, which is analogous to the blasto-derma or germinative membrane; careful examination showed that there was no decidua. It is also important to mention that, besides the yelk, which was of a yellow colour and quite distinct, a fluid matter was found between the cortical and vitelline membranes, thus occupying precisely the same space as the albumen in the egg of the fowl. This distinguished zoölogist further remarks that the cortical membrane was certainly not the decidua, but that "it is certainly analogous to the outer tunic of the uterine ovum of the rabbit and bitch, which in them is gradually separated from the vitelline membrane by the imbibition of an albuminous fluid."

It is our own conviction that what is here proved of one of the lowest of the mammalia applies to the whole class. It is no slight indication that the Fallopian tube is a real secreting organ, and not a mere passage of transit, that its mucous membrane in all the mammalia in which we have examined it, including the human female, is highly elaborated, forming in the outer two-thirds of its extent, a multitude of projecting valves; it is further found that this canal presents, in its uterine portion, a peculiarity of structure as marked as that seen in the isthmus of the fowl, when contrasted with the upper part of the oviduct. Developments and modifications like these must be associated with action and modification of function; and justify the inference that, in all mammals, the ovarian ovum receives, in its passage through the tube, in the first place an albuminous matter or white, and subsequently a distinct envelope, the chorion. In considering this question, it must not be overlooked that, amidst various modifications and much confusion, the best observers, from De Graaf to Baer and Martin Barry, have distinctly found the egg, previous to its attachment to the uterus, enclosed in two membranes, of which the outer is affirmed to be the chorion and the inner the yelk-membrane, or as it is called in mammals, the zona pellucida. The conclusion then at which we have arrived is, that in all essential particulars, the ovum of mammalia is identical with that of oviparous animals; an inference which is not only interesting in itself, but more especially because it gives an assurance that, in the subsequent development, a similar kind of identity will be preserved.

Having stated what we believe to be the view that is most in harmony with the general constitution and economy of the ovum, it is necessary to notice Dr. Bischoff's researches relative to this point. In his former work on the process of development in the rabbit, to which a prize was awarded by the Berlin Academy of Sciences, this excellent observer states that the ovum, in its passage through the Fallopian tube, receives a distinct albumen, but he contends that there is no cortical membrane or chorion surrounding it. In the work now before us, the Professor says, "the egg of the bitch, according to my observations, acquires no white either in the oviduct or in the uterus; this well-ascertained fact distinguishes the ovum of this animal very remarkably from that of the rabbit, which, as I have shown, receives a very distinct layer of white in the oviduct, and possesses it also, in the beginning, in the uterus. In the rabbit this matter soon disappears, since it becomes blended with the zona pellucida and with this represents the outer membrane of the egg, which, in the dog, is formed by the zona alone. It is therefore clear that no particular importance should be attached to this production of the albumen; and, therefore, that this last can have no essential influence upon the formation of the ovum and its coverings."—(*Entwickelungsgeschichte des Hunde-eies*, p. 69.) As regards the formation of the chorion, Dr. Bischoff says, in another place, it results from the junction or blending together of the yelk-membrane with the peripheral part of the animal layer of the germinal membrane.—(*L. c.*, p. 122). We can only repeat our dissent from the correctness of this account, and especially that which attributes the chorion to the zona pellucida, which seems to be, like its homologous part in the bird's egg, the vitelline membrane, namely, merely a retentive bag, and not an active structure.

In proceeding to notice more particularly the work of Dr. Bischoff, we would in the first instance observe, that it is a treatise to which, at this particular time, great value must be attached; for, although much of the matter it contains is not novel, there are many facts recorded relating both to the earliest parts of the process, and to the subsequent changes taking place in the uterus, which bear upon some of the most obscure but essential phenomena connected with the history of development, and which, at this time, engage a large share of attention of physiologists. In the second chapter, the author enters upon one of these difficult questions, the mode, namely, in which the semen operates on the egg. He justly remarks, what a reference to the writings of the older physiologists will amply confirm, that until lately the greatest doubt still prevailed upon the most important questions relating to the impregnation of mammals. In the following passage this uncertainty is well stated :—" It was not known whether the formation and escape of the ova was or was not dependent upon coitus ; whether the semen of the male played a material, and what part ; whether this fluid penetrated to the ovary, and in that place fertilized the egg, or whether both first met in the uterus or in the oviduct ; whether conception took place at the instant of connexion or subsequently ; and what changes the ovum thereon experienced ; all these questions were variously answered, and for the sufficient reason that there were no certain facts upon which to rest the reply."—(*L. c.*, p. 13.) At the present moment most of these doubts have received their solution ; and it is not useless to add that this might long since have been accomplished, if physiologists would have been content to receive information from the marked and ample phenomena displayed in the animal kingdom.

We need say nothing on the first question raised ; because it is now ascertained that the maturation and discharge of the ova, are processes altogether independent of any influence communicated by the male ; on this subject the researches of Raciborski, Negrier, Girdwood, Dr. Lee and others leave scarcely any room for doubt. With respect to the influence of the semen, the majority of our readers will recal the vague and frequently absurd theories, which some few years ago were put forth in works and lectures. And yet many accurate experimentalists, among whom Spallanzani, J. Hunter, Haighton, &c. may be mentioned, had distinctly proved that the act of impregnation depended on the material contact of the semen with the ova ; a fact equally substantiated by the mode in which the egg is fertilized in fishes and batrachia by the direct application of the seminal fluid. But all these and many other exact observations were insufficient to subvert the old doctrines of the aura seminalis, general sympathy, and other equally vague notions ; " it was in fact," to borrow the language of our author, " impossible to clear up all these involved questions, so long as the egg itself contained in the ovarium was not known ; and since it has been discovered, no one has devoted a sufficiently careful and successful investigation to the subject."—(*L. c.* p. 13). It is well known that Dr. M. Barry advanced the theory, that when an ovum was fitted for impregnation, it was carried up to the part of the Graafian follicle next to the outer surface of the ovary ; that the vitelline membrane or the zona pellucida then in turn became attenuated, and that at length a fissure was formed in it, through which a spermatozoon penetrated to the germinal vesicle. Dr. Barry subsequently obtained the impregnated ova of a rabbit from the Fallopian tube, in which he supposed, as well as several other physiologists to whom the specimens were shown, that spermatozoa could be detected actually within the interior of the zona pellucida.

This view of the subject, which is supposed to be in harmony with the known method of impregnation in the plant, where it is said the granules of pollen are conveyed down the style into the ovulum, has been to some extent received in this country; but according to Dr. Bischoff it is founded on a deception. He says, "I have stated in my work on the Development of the Rabbit, and have so depicted it, that the egg of that animal whilst in the Fallopian tube is covered with spermatozoa. I have even often seen them moving actively." "I am now certain that Dr. Barry has mistaken spermatozoa situated *upon* the ovum, for the same bodies *within* it." Those who have themselves made observations upon ova taken from the tube, are aware of the great difficulty of deciding whether the spermatic animalculæ, which are, unquestionably, *in immediate contact with the egg*, are without or within the zona pellucida, and will therefore be inclined, for the present, to suspend their judgment upon this deeply interesting point of embryology. It is, however, most important to know that, whatever may be the solution of this question, it is well ascertained that the seminal fluid, as indicated by the presence of its animalcules, penetrates as far as to the ovarium, upon which it has been seen by Martin Barry and Bischoff in the rabbit. In the work of the latter, now before us, the following conclusions are given, after numerous observations, with respect to the dog:

"1. The semen penetrates at the instant of copulation through the os uteri into its cavity, and even as far as the furthest point of its horn.

"2. The semen in the subsequent hours penetrates also into the Fallopian tube, and can thus reach the surface of the ovary.

"3. The semen comes, in every instance, into material contact with the ova, and impregnation is effected by means of a material reciprocal action between the semen and the egg.

"Impregnation never occurs at the moment of copulation, but at a later time, to be hereafter specified."—*L. c.* p. 16.

Although there are no sufficient data at present, to enable the physiologist to ascertain the precise influence which the semen exerts on the ovum, it will still be useful briefly to consider this question, which however viewed is full of mystery. That the effect produced is most marked, is more particularly shown by the history of hybrids, and in a less striking degree by those physical peculiarities or resemblances which are impressed on the new being by the male parent: but on these points we do not propose to touch. There is no doubt that the semen operates upon the contents of the germinal vesicle, and whatever may be the subsequent (morphological) changes therein induced, the earliest ones seem to be of a chemical character; an inference which receives some support from the fact that, whereas the ovule of the plant prior to impregnation contains only starchy matter, it presents subsequently to that phenomenon another organic principle forming the walls of the newly-produced cells. This view corresponds with that advanced by Professor Bischoff, to the effect that the operation of the semen upon the egg is in the first instance of a chemical nature: and he further believes, with Valisneri, Bory St. Vincent, Valentin and others, that the object of the spermatozoa is to maintain by their motion the easily-changed composition of the semen.

As to the time and place of impregnation they are both subject to variation. The author concludes that, in the bitch, the ovum is capable of being impregnated, during the whole of the interval occupied in its transmission from the ovarium as far as to the end of the Fallopian tube, that is to say, from six to eight days; but when the egg has reached the uterus, it is no longer susceptible of the influence of the semen, inasmuch as the process of development commences, after conception, in the lower part of the tube. In support of this position, it is an interesting fact that in all animals subject to periodical venereal excitement or *heat*, this entirely ceases when the ova have arrived at the uterus. With respect to the *place* of impregnation, the principal point to determine is whether it is possible for ova to be impregnated whilst in the ovarium. If the records of extra-uterine conception in the human subject are to be relied on, this may take place; for cases are referred to, among others, by J. Hunter, of ovarian pregnancy.* It must, however, be confessed that the excellent researches of Prevost and Dumas, joined to the now ascertained fact that the actual contact of the semen is required, have created a rather general belief that the ovum escapes from the ovarium in consequence of the act of copulation, and that the semen coming in contact with it, either in the tube or in the uterus, impregnation takes place in one or other of these organs, and nowhere else. This opinion has been more positively affirmed in a late work published by M. Pouchet (Theorie Positive de la Fécondation des Mammifères, Paris, 1842). This writer says that physical obstacles are opposed in mammalia to the contact of the semen with ova still contained in the Graafian follicles; that assuredly ovarian pregnancies, properly so called, do not exist; and that, as fecundation in mammals, takes place normally in the uterus, abdominal and tubular pregnancies do not indicate that impregnation is effected normally in the ovary.

The observations of Dr. Bischoff on this question are so important and exact, that no apology is required for laying them before our readers in the author's own words. After stating that he has repeatedly found, in the rabbit and dog, spermatozoa in different parts of the Fallopian tube, between the fimbriæ, and upon the ovarium, this distinguished physiologist remarks:

"All theoretical objections are entirely disproved by these direct observations. I have also further shown that there is not the least impediment to the penetration of the spermatozoa into and entirely through the Fallopian tube, the movements of those bodies and the contraction of the tube itself being quite competent to effect such a result. Again, as to the assumed difficulty of the impregnation of an ovum whilst still in the ovarium, they do not exist. I have expressed my conviction that the fluid portion of the semen is the fructifying part, and that the smallest amount of it is sufficient to produce the effect. There is nothing therefore in the way to prevent the semen penetrating through the coverings of the ovary and of the Graafian follicle, till it reaches the ovum; especially if it be recollected that all these envelopes, at the moment when the

---

* Hunter admits three forms of extra-uterine conception, according to the situation of the fœtus, namely, ovarian, tubular, and abdominal; it is evident, however, that the latter is owing to the escape of the ovum by rupture or absorption from one or other of the former situations. Hunter justly remarks on the obscurity attending these cases. They are, he says, "extraordinary and seldom happen, and when they do occur, are often attended with so many hindrances to critical investigation, as hardly to allow of thorough or satisfactory information."

ovum is on the point of escaping, are thinned to the uttermost. It is, therefore, certain that eggs still in the ovarium can be impregnated; but this does not altogether prove the possibility of their becoming developed in the ovary, or of ovarian pregnancy, a phenomenon which seems to rest on incorrect and inaccurate observations."

He then adds that ovarian conception is, however, only the exception; for as the ova quit the ovarium, not in consequence of the influence of the semen, but of their being fully developed, and as in general this happens before the seminal fluid has had time to reach the ovary, the rule is that "*the ova and the semen meet together in the Fallopian tube, and that here impregnation takes place.*"—*L. c.* p. 30.

In this extract we have no doubt the phenomenon relating to the seat of impregnation are correctly set forth, excepting that it is with us a question, whether, in ovarian conception, the semen does not come into direct contact with the ovum, the Graafian follicle, whilst grasped by the fimbriæ of the tube, being as usual ruptured, but the egg, subsequently to the act of fertilization, being by some unknown cause retained within the ovi sac, and there becoming developed.

Although, as we have said, it is not our object to trace the process of development, it will be desirable to notice some of those parts of it, concerning which at present there prevails much uncertainty. One of these relates to the first changes that ensue in the ovum consequent upon its fecundation; and this should be a subject neither for surprize nor discouragement when the minute size of the objects to be examined are recalled to mind, the ovum of the bitch having, when it quits the Graafian follicle, a diameter not exceeding the $\frac{1}{10}$ or $\frac{1}{15}$ of a Paris line, whilst that of the germinal vesicle is pretty constantly in the dog $\frac{1}{50}$, and of its macula $\frac{1}{200}$ of a line. Those of our readers who have kept up with the progress of modern embryology, will recollect that all investigators are agreed in attributing to the minute vesicula germinativa, and even to its spot or nucleus, some predominating influence in the first acts of development: it is even thought and apparently proved, that the basis of the embryo is produced by the direct operation of this fundamental element of the ovum. But, although there is this accord with reference to the supreme importance of the Purkinjean vesicle, there is the greatest discrepancy as to its mode of action. These conflicting views may, however, be referred to two hypotheses: according to one, and it is that which, resting on the deeply interesting researches of Dr. M. Barry, is rather generally adopted in this country, the vesicle receiving the direct influence of the semen (according to Barry a spermatozoon penetrates into it), takes on a complex series of cell-formations, determined especially by the reproductive powers of the macula germinativa, and which ends at length in forming the first rudiments of the germ, so that, in this theory, the vesicle is not only the determining cause of the primary acts of development, but also the seat of them, the yelk thus performing the merely subordinate part of furnishing by endosmose the crude nutritious matter.

According to the second, more generally received, and, as we believe, the true hypothesis, the vesicula either is ruptured or dissolved as the immediate and necessary result of a fruitful copulation; it thus disappears as a vesicle, though the macula or nucleus germinativus may remain intact, and play a very active part in the subsequent evolutive process. This is the account of the matter to which Professor Bischoff inclines in this and his earlier works; he having always contended, sometimes we think with unjust asperity, against Dr. Barry's peculiar views. The series of actions consequent upon this rupture of the germinal vesicle has been variously regarded; but there is much concurrent testimony, and we allude herein more particularly to the earlier researches of Prevost and Dumas, to the subsequent ones of Rusconi, Baer, Rathke, and Siebold, and to the still later ones of Kölliker, Bagge, Vogt and others on the remarkable process called *the cleaving of the yelk;* there is we say much concurrent evidence to show that the way in which the contents of the vesicle conduce to development is by determining the subdivision of the vitellus into minute segments or globules which are ultimately converted into nucleated cells, and that these united together constitute the germinal membrane or true rudiment of the embryo. Thus it is found that, after impregnation, the yelk has in its centre a single cell (the first embryonic cell according to Kölliker): this cell next divides into two secondary cells, and at the same time, and apparently as a consequence, the yelk also breaks up into two halves; and this process of subdivision goes on in arithmetical progression, till at last a mulberry-shaped mass is produced lying of course within the zona pellucida or vitelline membrane. In the bitch, according to the author, the ovum at the end of the Fallopian tube has between 16 and 32 of these segments; and in the centre of each he found a very delicate vesicle, powerfully refracting the light.—(*L. c.* p. 44). There has been much difference of opinion as to the exact relation which the segments of the yelk and the central bright vesicle have to the cell-formation; but, without dwelling upon this involved point of minute anatomy, it will suffice to state the general conclusions of Professor Bischoff on this subject. He says "these yelk-globules are not cells, but an agglomeration of yelk-granules without an envelope; each contains in its interior a transparent vesicle, similar to a fat vesicle, but without a nucleus, As to what determines this process of cleaving, and what is the source of the transparent vesicle, nothing is still certainly known; but it appears that the cleaving of the yelk and of its segments, is dependent upon these vesicles, and that these take their origin either from the germinal vesicle or from its nucleus." "In the uterus, the ovum has at first still the same appearance as in the oviduct, and the subdivision of the yelk still proceeds. But now the yelk-globules, continually diminishing in size, transform themselves into cells by becoming surrounded with a delicate membrane. The nuclei of these cells are the transparent vesicles placed in the centre of the globules. These cells arising out of the yelk-globules rapidly join together, and, their number still increasing, represent, by becoming flattened and blended together, a very delicate membrane lying close beneath the zona pellucida, which thus appears as a vesicle, called by me the *vesicula germinativa.*"—*L. c.* p. 119, 120.

Such, then, are the earliest changes in the fecundated ovum, by means of which the first rudiment of the embryo is laid down in the form of a delicate cell tissue, commonly called the germinal membrane, but as we have just seen named by the author, germinal vesicle. We shall not pursue the subsequent changes of development, but proceed to notice what has more interest for the medical practitioner—the mode of attachment of the ovum to the uterus, and the formation of the placenta.

There can be no hesitation in affirming that, of all the branches of embryology, by far the most difficult in its investigation and the most involved in its description, is that which concerns the connexion existing between the uterus and the fœtus; and, notwithstanding the numerous and valuable contributions made of late years to this important subject, if we may judge from some of the publications that have appeared in this country, and from conversation with many of our professional brethren, very vague notions still prevail even among professed accoucheurs. The time, however, is arrived when the obscurity and contradictions in which the description of the placenta has been involved, may be all but completely removed; and it will be our object, with the aid of the recent researches of Professors E. H. Weber and Bischoff, combined with those of other observers, to give a clear and succinct account of this matter. To do this it will, as in all similar cases, be necessary to consider the earliest changes in the uterus consequent upon conception, and the first relations established between that organ and the ovum. It is well known that, as the result of impregnation, there is formed in the uterus the production called *decidua;* and that, under this name, two substances are comprised, one lining the inner surface of the uterus being called the decidua vera s. uteri, and another investing the ovum, called the decidua reflexa. Now, until the researches to which we shall immediately refer, these two productions were generally supposed to be both formed by a coagulated lymph secreted by the mucous coat of the uterus; but it is a point of primary importance to know that this is not the fact. The two tunicæ deciduæ are perfectly distinct structures; one, the decidua vera, is nothing else than the *developed and thickened mucous membrane of the uterus;* whilst the other is unquestionably a *true secretion,* derived in all probability from the uterine glands. For the first positive step in the right direction, and by which a beginning was made for the reversal of the generally received Hunterian doctrine on the production of the decidua, we are certainly indebted to Professor E. H. Weber, who, in his edition of Hildebrant's Anatomy, minutely and accurately described in ruminants the glands of the mucous membrane of the uterus which he had already seen in the uterus of a woman, impregnated seven days previous to death, and which, on account of their form, he named "*glandulæ utriculares.*" He also especially pointed out the manner in which the cells of the chorion take up the yellowish fluid poured out by these flask-shaped glands.

The existence of these most important glands seems to be general among mammalia: thus they have been discovered by Eschricht in the porpoise and cat; in the rabbit by Weber; in the bitch by Dr. Sharpey; and in the human uterus by Professor Weber, and independently, by Dr. Sharpey, to whose admirable description in Dr. Baly's translation of Müller's Physiology we have much pleasure in referring our readers. On the present occasion we shall principally avail ourselves of the recent observations of Professors Weber and Bischoff, which appeared in Müller's Archives for 1846.

Professor Weber states that, after conception, the mucous membrane of the human uterus becomes soft, and, by degrees, attains a thickness of two or three lines: *it is this structure which forms the tunica decidua uteri.* This change depends upon the increase partly of the vascular and partly of the non-vascular layer of this membrane or its epithelium; that is to say, the blood-vessels of the mucous membrane of the uterine glands become enlarged, and, at the same time, an abundant formation of white epithelial cells, partly nucleated, takes place. The utricular glands thus enlarged are two or three lines in length; they run like the gastric glands towards the inner or free surface of the mucous membrane, and there terminate by orifices, which have long been known as existing in the tunica decidua, to which they give a cribriform appearance.

In the dog and cat the uterine glands, which in these animals are of two kinds, simple and large or branched glands, enlarge themselves in like manner at the place where the placenta subsequently appears; the smaller ones in their whole length, but the larger branched ones only towards their orifices, where they become dilated into a number of sacks or pouches, around the walls of which the uterine blood-vessels are dispersed, and which, as they enlarge, push as it were against the lining membrane, carry this before them in a complex manner, and thus become enveloped by it in the same way as the large intestine is covered by the peritoneum.

The fact that the decidua vera is composed actually of the developed uterine glands, and not, as the Hunterian doctrine taught, of an exuded fibrine, must now be regarded as firmly established; and this, which is the clue to the whole formation of the placenta, requires us in the next place to consider what is the nature of the connexion established between the outer membrane of the ovum, the chorion, and the so-called deciduous membrane. In the ruminantia there is apparently afforded the type of what, under different modifications, takes place in all mammalia: in these animals it is found that the tufts and villi of the chorion are received into the glandular tubular canals of the maternal cotyledons, just as the fingers are introduced into a glove. The connexion consists of intimate contact, not of union, and so the tufts of the fœtal cotyledons can be easily drawn out—in the gravid uterus of the cow, for example—from the sheath of the maternal ones, when it is seen that they were bathed in a milky kind of liquid. A similar relation, in principle, has been detected by Drs. Sharpey and Weber in the bitch; the latter thus describes it. "The tufts of the chorion, which carry the embryonic capillary vascular plexus of the umbilical vessels, grow, as it were, into the expanded openings of the uterine glands; fill out the expanded part of these glandular flasks; and, insinuating themselves into all their folds and tufts, grow with them, and thus form together a particular membrane, which alone possesses embryonic vessels. In this process of development, the part of the membrane derived from the walls of the uterine glands becomes probably thinned by absorption." This important and elaborate process is confined to the dilated pouches of the uterine glands, as the tufts of the chorion do not seem to penetrate into their tubular portion, nor into their cæcal extremities.

The exact structure of the zonular placenta of the bitch produced in the way just described, is thus explained:—the whole placenta is penetrated or traversed throughout by a coarse network of tortuous capillary vessels, carrying the maternal blood, and having the very large diameter of from about $\frac{1}{52}$ to $\frac{1}{63}$ Paris line; these vessels are each of them overlaid or covered by a membrane, which gives transmission to a very rich network of extremely minute embryonic vessels, the diameter of which being $\frac{1}{173}$ to $\frac{1}{234}$ of a Paris line, is three times smaller (giving an area nine times smaller) than that of the maternal blood-vessels which are drawn over them. Thus it happens that the embryonic blood contained in rich vascular retia, flows over the surface of the large canals carrying the maternal blood, but evidently in such a manner that the two kinds of vessels and their contents do not communicate together; they therefore only come into indirect but most extensive relation with each other, the disposition being similar to that of the small branches of the air-tubes with the rich networks of the pulmonary capillaries displayed over them.—*Müller's Archiv.*

These investigations into the structure of the placenta in the dog are so important in themselves, and supply so many of the voids at present existing in the human formation, that we are tempted to extract the very clear account given by Professor Bischoff as the result of his researches.

"Until the ovum has acquired the size of from 2 to 2½ lines, and when it has still no villi on its surface, it lies quite free in the uterus, and no other change is seen in the mucous membrane of the latter organ, except that it is more vascular, turgescent and villous than usual. But when the ovum has attained the above size, the mucous membrane, at the place where the placenta is subsequently formed, rapidly develops itself, and soon forms a distinct elevation on its inner surface. If the free surface of this be now carefully observed, there will be seen, even with the naked eye, a great number of small apertures, and soon when the ovum with its tuft and the above elevation have increased in size, we can perceive that the tufts of the chorion project into these apertures: (this disposition is beautifully illustrated in the accompanying plates, fig. 48, A.) In the beginning, the tufts of the chorion can, after a slight maceration, be readily drawn out of the apertures; in a short time, however, this cannot be so easily effected, but" (and to this point we would direct the particular attention of our readers) "we can now much more readily accomplish a separation of the whole swollen part of the mucous membrane of the uterus, which remains attached to the ovum; the separation of this layer takes place so much the easier as the egg is more developed. Thus is formed the placenta of the dog. If a section of the mucous membrane be made at the zonular swollen place above described, it will be found that the swelling consists partly of a succulent infiltration of the whole tissue, but more especially of the above described uterine glands greatly developed: the small apertures noticed above as being seen on the free surface are the orifices of these glands, into which sink the tufts of the chorion."

Although the subsequent changes are difficult to decipher with accuracy there is no room to doubt that they consist essentially of the continued progress of the same disposition; that is to say, the uterine glands increase more and more, and with them the tufts of the chorion, which, sending off numerous lateral branches and offsets, project into them as into sheaths. The umbilical blood-vessels of the fœtus ramify in the tufts, the arteries passing over into the veins in loops; and in a similar manner the blood-vessels of the mother expand themselves between the uterine glands, a network of capillaries forming the connexion between the uterine arteries and veins. The whole forms the zonular placenta, in which therefore can be distinguished a maternal part, formed especially of the greatly developed uterine glands and uterine vessels; and of a fœtal part composed of the tufts of the chorion and the umbilical vessels ramifying within them. The maternal and fœtal blood pass by or over each other in capillary streams, but without actually intermingling; the tufts of the chorion, it is to be observed, are not sunk into the sinuses of the uterine veins, but into the tortuous and greatly enlarged uterine glands. Both parts of the placenta are subsequently so intimately blended together, without, however, any departure from the fundamental arrangement, that they cannot be separated; but the maternal part may be detached by laceration of the connecting vessels and fibres of the developed part of the uterine glands; in fact, the uterus in this place is stripped of its membrane, which, with its glands, is afterwards regenerated.—(*Entwickelungsgeschichte,* &c. pp. 114, 115).

In this description, we have directed attention to the important circumstance that, after development has advanced to a certain extent, the decidua uteri readily tears off. This explains what happens in the case of human gestation, and especially shows how the decidua uteri may be raised in dissection from the uterus, as if it were a layer distinct from the mucous membrane.

In proceeding to the investigation of the human placenta, there is doubtless much more difficulty in analysing its minute structure, though the main facts are fully determined. There are some important points in which it strongly contrasts with the placenta of mammalia; and we are ourselves of opinion that, taking these differences into consideration, it may be predicted that ultimately the mode of formation and constitution will be found to be essentially the same in man and animals. The principal differences to which we allude are these: that in the human female there is an additional element concerned, the decidua reflexa, which has no certain existence in animals; and secondly, that in man, the fœtus is larger than in any animal having a single placenta, that has hitherto been investigated, a circumstance which must modify the character of the placenta, though it may not alter its essential constitution. In the valuable contribution to which we have alluded, Professor Weber mentions several circumstances, from which he seems to doubt whether the human placenta is formed according to the same principles as that of the cat and dog: the most important of these circumstances we shall now quote, both because they bear upon the point in question and also because they convey several important facts connected with the structure of the placenta. The peculiar enlargement of the ducts of the uterine glands into the wide and folded pouches already described, has not yet been seen by Dr. Weber in the human female; nor in a gravid uterus of the tenth week could he discover that the branched tufts of the chorion had pushed into the openings of the utricular glands; and moreover, the simple figure of the utriculi of the human uterine glands did not correspond to the infinitely ramified and divided tufts of the chorion. It cannot, therefore, be assumed as proved, that in man the tufts of the chorion grow into the utriculi of the uterine glands, as they certainly do in the dog. Again, as man is distinguished by exclusively having a decidua reflexa, so a difference may exist as to the nature and formation of the placenta. Several other subordinate distinctions are also pointed out; such as, that the placenta uterina of the human female is distinguished in this—that the coarse vascular rete carrying the maternal blood, and which penetrates through the whole placenta, consists of canals having a diameter fifteen times larger than the capillaries that bring the maternal blood into the placenta of the dog, whilst the walls are much thinner: that the other element of the placenta, the tufts of the chorion, carrying a very rich rete of minute embryonic capillary vessels, form in the dog membranes and folds, in man, on the contrary, small trees with cylindrical branches and twigs; that in the human placenta there are, in the usual sense of the word, no capillary vessels, but only vessels of $\frac{1}{4}$ to $\frac{3}{4}$ of a line in diameter (colossal capillaries or veins), and that the arterial branches $\frac{1}{4}$ to $\frac{3}{4}$ line in diameter, which conduct the maternal blood out of the uterus into the placenta, do not divide themselves repeatedly into branches, but form, in their passage into the placenta, a coil (*glomus arteriosus*), which consists of a single artery twisted here and there, and which at length prolongs itself directly into the network of those colossal capillary vessels or veins which penetrate through the whole placenta: lastly, that whilst it happens both in man and in the dog that the vessels carrying the maternal blood come into intimate contact with those carrying the embryonic blood, yet, in the latter, it happens that the vessels carrying the maternal blood into the placenta are individually developed between the membranes and folds of the tufts of the chorion, and are covered by them; in the former, on the contrary, the branches and fibres of the tufts of the chorion are covered by and developed in the walls of the very wide and thin-walled maternal blood-vessels, which, filling up the intervals between them, insinuate themselves on and so surround the tufts. Professor Weber finishes this summary by remarking that, if it should be shown hereafter that the tufts of the chorion in man, as in the dog, grow into the utriculi of the uterine glands, and thus fill them up, it would thence follow that the branches and terminal fibres of those tufts, would acquire a thin and adherent covering, derived from the walls of the uterine glands.—*Müller's Archives*, 1846, p. 424.

In opposition to the objections set forth by Weber, but evidently without any certain conviction of their truth, we would again refer our readers to the account of Dr. Sharpey, and especially to the figures illustrative of the uterine glands of the bitch, and of the same organs in the human subject. We shall also now append the most recent views of Professor Bischoff on this important point. In the work before alluded to (*Müller's Archives* for 1846, p. 111), a very instructive case of early pregnancy is related by this admirable observer.

From the facts stated, there is no doubt that impregnation had occurred within 14 or 21 days before death. The case was that of a married woman, aged 31, who had borne eight children, and who destroyed herself by drowning.

"The inner surface of the cavity of the uterus had an appearance quite different from the usual one, and which became very distinct when the organ was placed in water; it had, namely, a very delicate and apparently tufted condition, and this was particularly distinct upon the cut borders. The surface itself, if viewed from above, appeared as if finely perforated; or rather, as if closely covered or beset with small white points, which, upon the section, were perceptible as the free ends of the apparently white tufts. These little tufts, however, were not such in reality; for, firstly, they were all connected together by means of a semi-transparent mass; and further, it was easy for a person experienced in these investigations to perceive, by employing magnifying powers, that these apparent tufts were small glandular canals or pouches (*drüsenschlauche*) 1½ to 2 Paris lines long. The analogous formations of the dog, the cow, the sow, &c. being known, I could not remain in any doubt, their nature was apparent at the first glance—they were the same uterine glandular canals which I had sought for in vain in the unimpregnated condition (of the human uterus), but which here showed themselves quite clearly and distinctly, even to the naked eye." "Now, as I have already entirely convinced myself that in the dog (herein agreeing with Sharpey) these glandular canals, expanded into flask-shaped pouches, take up the tufts of the chorion, to form together with these in the rapid progress of development, the so-called decidua in the bitch's ovum, so I hold it to be no longer doubtful that, in the human female, a precisely similar disposition takes place. These glands seem to be very small and indistinct in the unimpregnated state; but after conception they grow rapidly, and an exudation also occurring from the surface of the uterus, they in a manner grow into this exudation, and the two together then form the decidua vera; and, at that part where, owing to the application of the allantois, the tufts of the chorion become further developed, the placenta. The decidua, therefore, is in fact, if not exactly the membrana, at least the stratum uteri internum evolutum; and, as such, a formation is present, partly as a product of development and partly as a new formation. At birth, a real casting off of the inner (mucous) layer of the uterus takes place; but it is to be presumed that the fundamental part of the glandular canals, namely, their cæcal extremities, which project against the fibrous coat, are left behind."—*L. c.*

In this account there is one circumstance to which attention should be particularly directed, as it will explain one of the many sources of error by which the formation of the decidua vera has become so involved and obscured. We allude to the circumstance, that when the uterine glands become enlarged, they give to the free surface of the mucous membrane of the uterus, what would not have been expected *à priori*, a peculiar *tufted appearance*, and which has consequently been often mistaken for true villi. For the rest, it is very satisfactory to find the important researches of Weber and Sharpey concerning the uterine glands, thus confirmed by an anatomist, who probably, owing to his extended and successful researches, was the best qualified in Europe to offer an opinion.

There is still some uncertainty concerning the nature of the decidua reflexa; this is of the less consequence, as it is altogether a secondary part, and is absent in animals. We have already said that it has an origin totally distinct from that of the decidua vera. Dr. Sharpey suggests, without wishing to affirm anything positively, that it may consist of exuded lymph, which covers the minute ovum on its entrance into the uterus, either entirely or on that part of its surface which does not adhere to the uterus. Mr. Goodsir, who has satisfied himself that the decidua reflexa is a distinct formation, offers an explanation of its origin in accordance with his views of the process of nutritive absorption in the alimentary canal; namely, that this covering, which he proposes therefore to call the *cellular* decidua, is formed of cells passing off from the uterine glands, and which surround the ovum when it enters the cavity of the uterus. Connected with this view of the origin of the d. reflexa, he has a theory that this structure, by the powers of its active cells, nourishes the ovum after this has exhausted the supply provided in the ovary, and that it thus performs the same part in the gestation of the mammal, which, in the egg of the oviparous animal, is effected by the albumen.

In the plates illustrative of development published by Dr. Erdl, there is a scheme representing the formation of the decidua reflexa (*Erster Band, Zweiter Theil,* Tab. 2, fig. 8), which is altogether too mechanical; and in the brief description of the plates, the author gives what is certainly an erroneous account of the production of the above layer, inasmuch as he states it to be nothing else than the prolongation over the ovum of that part of the decidua uteri which he affirms, but again erroneously, extended, prior to the descent of the ovum, over the orifice of the Fallopian tube. Upon this point Dr. Bischoff properly remarks, that as we are speaking of a most minute body, of the 1-12th of a line in diameter, all mechanical explanations of the way in which the ovulum becomes covered by the decidua reflexa, must fail of necessity; he himself considers the question to be undecided, and to require further research.

We have been led further into detail than we had designed; but as no true or clear idea of the structure of the placenta can be formed, without the relations of the ovum and uterus are definitely made out, it is hoped this consideration will prove a sufficient apology for the extent of the foregoing observations. Those who have carefully followed the account now given, will find a cloud of doubts and difficulties removed; and by comparing especially the critical observations of Weber upon the reciprocal relations of the various maternal and embryonic vessels and membranes contained in the placenta, with the accounts given by those writers, who have discovered many isolated facts connected with this body, often, however, mixed up with error, much that before was confused, apparently even contradictory, and therefore most unsatisfory, will become clear and comprehensive. The account of J. Hunter is certainly the most important of all the older histories of the placenta; and but for one pervading error, would probably long since have conducted his successors to the true anatomy. The error to which we allude is that Hunter, led away by his great principle concerning the effusion and organization of fibrin, regarded the decidua (both the vera and reflexa) as formed by an effusion of coagulable lymph, and which, like the effused fibrin poured out into any of the cavities of the body consequent upon the introduction of an extraneous living part, a comparison made by Hunter, became subsequently organised and vascular by the prolongation of vessels from the inner surface of the uterus.* Another error also occurs in this account, namely, that the ma-

* It is interesting to compare the account given by Hunter of the first changes induced in the uterus as the result of conception, with the late researches which we have noticed above. In a case where impregnation had occurred within one month before death, the inner surface was found covered with a pulpy substance in its thickest part forming a layer ½ line thick, and "evidently formed by coagulated blood;" this substance was continued across the cervix, thus closing the cavity of the uterus, and also extended some way into the Fallopian tubes. "When the inner surface of the cavity of the uterus was examined with a magnifying power, it was found extremely vascular and dotted with innumerable whitish spots, too small to be seen by the naked eye." There is no doubt that these "whitish spots" were the orifices of the enlarged uterine glands filled with an abundant epithelium. We have quoted the authority and description of John Hunter, without at all alluding to the dispute between him and Dr. W. Hunter as to priority of discovery.

ternal blood carried into the placenta by the curling arteries was there deposited in cells, from which it was said to be returned by channels ultimately leading into the uterine sinuses. This view was finally adopted by Mr. Owen, after he had for a time received as correct Dr. R. Lee's account. He found, on examining a gravid uterus of the ninth month, that the uterine veins were continuous with wide channels, passing obliquely through the decidua into the placenta, which "decidual canals," as they are here called, became diffused through the fine spongy and cellular substance which every where surrounds and supports the fœtal capillaries, and at length penetrated to the surface of the placenta. This distinguished physiologist concluded that "the placental intercommunication between the fœtus and the mother, in the human subject and the quadrumana, is carried on by the contact of the fœtal capillaries with maternal extravasated blood; while in ruminants, the mare and the sow, it takes place by the apposition of capillaries to capillaries, and the two parts of the placenta, namely the fœtal and maternal, can be separated. In the feræ and rodentia there appears to be an intermediate structure."

The first correct view of the vascular arrangement of the placenta was taken by E. H. Weber, so long ago as 1832; and as the account given by that admirable anatomist is in itself essentially true, and has laid the foundation of all later researches, we are desirous of briefly submitting it to the notice of our readers. He first accurately describes the entrance of the uterine arteries and veins into the placenta, by passing obliquely through the decidua vera, thus confirming the fundamental fact ascertained by Hunter, but which has at various times been called in question. He notices the fact that, the coats of these maternal vessels become, on entering, extremely thin, and are therefore easily lacerated. The true relations existing between the maternal and the fœtal portions of the placenta are then, for the first time, thus accurately determined:—"The fœtal part of the placenta consists, in man, of the multitudinous arborescent tufts of the chorion, which project into the canals of the widely-dilated veins filled with the maternal blood; these latter vessels pass from the inner surface of the uterus through the decidua vera, and penetrate throughout between the tufts of the fœtal portion of the placenta, and thus form a large network." After pointing out the appearances that doubtless deceived Hunter and his successors, as to the existence of *cells*, namely, that the uterine veins, where lodged between the tufts of the chorion, losing their cylindrical form, owing partly to the extreme tenuity of their walls and partly to their disposition, have the aspect rather of interstices and passages than of veins, Professor Weber thus explains the true structure: "it occasionally happens, however, that we are so fortunate as to find a place where it can be seen in what manner the veins are related to the tufts. At the borders, namely, of the placenta and sometimes in its substance, entering uterine veins are found, into the interior or cavities of which a small tuft of the fœtal portion of the placenta here and there projects, whilst the vein still has, in other respects, entirely the characters of a definitely limited canal with smooth internal parietes. We can in such places convince ourselves, that these tufts of the placenta fœtalis, thus projecting into the interior of the veins, do not pass through an actual perforation in the vein, but that the inner extremely thin venous membrane at the place where the tuft pushes itself in, is carried before it into the cavity of the vein, and that this projected part (of the lining membrane) is carried over the tuft and each of its individual fringes; or, in other words, that each tuft projecting into the vein fills up the pushed-forward, inner venous membrane, and that in this manner this latter is carried over the fringes of the tufts."

After again insisting upon these relations of the maternal and fœtal portions of the placenta, and precisely defining the whole structure, Weber gives the following summary of the *modus operandi* of this complex organization.

"The physiological actions of the human placenta appear then to consist in this—that the large stream of the embryonic blood is so conducted over the still larger current of the maternal blood, as that every blood-corpuscle of the fœtus, whilst it circulates through the placenta, comes into a very intimate but indirect contact with the blood of the mother for some considerable time. This is effected in consequence of the blood-stream of the embryo being so divided by traversing an infinite number of minute canals, that only a single series of corpuscles can pass along, whilst the maternal stream flows in very wide thin-walled passages, into which the tufts of the fœtal placenta project, and are bathed by the maternal blood flowing over them. Now, since the blood of the fœtus flows through the fine, hair-like extremities of these tufts, it may be presumed that it can attract through the extremely thin and moist parietes of the fine and elongated capillary vessels, certain substances contained in the maternal blood; and, on the other hand, that the maternal current of blood can doubtless also effect, through the attenuated parietes of its containing canals, an attraction or absorption of certain substances existing in the fœtal blood.—(*E. H. Weber's edition of Hildebrant's Hand. der Anat. Band,* IV. p. 496-499.)

The researches we have thus cursorily explained, illustrate so perfectly and minutely the intimate texture of the placenta, that, combined with the equally important and original observations of the same author upon the uterine glands and the arrangement of the capillaries of the umbilical blood-vessels, they have left little more to be determined. They entirely explode the Hunterian doctrine of the maternal blood being extravasated or deposited by the curling arteries in the so-called "cells" of the placenta; they reveal the exact disposition of the chorion-tufts and of their contained blood-vessels; they do away with all the vague ideas of vessels with open mouths; they disprove the agency of lymphatics; and, finally, they explain the physiological phenomena connected with the introduction of nutritious matter into the system of the embryo. The term "cells of the placenta," indicating thereby extra-vascular cavities, has been so frequently used in descriptions of this organ, and in this country even of late years by writers who are looked up to as high authorities in this branch of medical science, that it is desirable distinctly to state that no such "cells" exist. It may be affirmed as a fixed law of the circulation, that the blood never quits its containing channels *en masse;* that is to say, it is never extravasated. The principal cause of the errors which have so constantly prevailed upon this point among writers unacquainted with microscopic and structural anatomy, are firstly, that where blood-vessels, and especially the veins, are either removed from external pressure, as in the case of the bones, or are connected with functions demanding a ready passage to and from the contained blood, their parietes become extremely attenuated, and are therefore often overlooked; and, secondly, that when such vessels are artificially injected, especially when this is rudely done, as is usually the case, their delicate membranes are torn through, and a deceptive appearance of cells is thus produced.

The later inquiries of Dr. Reid and Mr. Goodsir have confirmed the account of Weber, and have thrown additional light on the minute relations of the maternal and fœtal vessels and membranes. The observations of the latter gentleman are particularly interesting; and although several of his physiological inferences are to a great extent speculative, his paper should be carefully considered by all who desire to become thoroughly acquainted with the subject to which it relates.—*See Med. Chir. Rev.*, July 1845.

As additional evidence of the correctness of Professor Weber's views, is still desirable, we may take this opportunity of stating that, we have lately had an opportunity of examining two gravid uteri of about the fifth and sixth month respectively, and in which the placentæ having been previously minutely injected both from the maternal and fœtal vessels, were favourably displayed. We were thus enabled distinctly to follow the uterine veins into the placenta, the passage taking place opposite to the interlobular spaces. Where quitting the fibrous coat of the uterus just at the point of entrance into the placenta, the walls of these enlarged veins become extremely thin and most liable to laceration, a circumstance which is doubtless a principal cause of the uncertainty that has prevailed upon this disposition. Just where the uterine veins had got to the placenta, tufts of the chorion were seen projecting into their cavities, as described by Weber and Reid ; but no tufts were in either case seen pushing into the veins of the walls of the uterus, a disposition noticed by the latter observer. An injection of size having been employed, the curling uterine arteries could not be traced very satisfactorily into the placenta; but it was yet evident that at several points this transit took place. The maternal vessels having been filled in one of the specimens with red and the fœtal vessels with a yellow injection, the two orders of tubes could be distinctly followed ; and in this way it was found, what other observers have noticed, that the uterine vessels penetrated completely to the fœtal surface of the placenta, and the umbilical vessels, as above stated, as far as to where the placenta came in contact with the fibrous coat of the uterus. A clean vertical section through the fundus uteri, displayed very beautifully the remarkable mechanism of the uterine veins for preventing hæmorrhage on the separation of the placenta; the free inter-communications of these vessels being provided with valve-like processes of the fibrous substance of the uterus, produce the same effect as if each of the uterine veins had been divided into a numerous series of chambers provided with muscular, contractile valves, and thus capable of being completely closed. This disposition becomes more and more developed, as the veins approach the inner surface of the uterus, or the place where, in parturition, they are torn across; and it thus happens, as Professor Owen has so well described, that on the forcible contraction of the organ, subsequent to the separation of the placenta, the whole of this complex valvular apparatus comes into play, and, by compressing the enormously dilated and now ruptured veins, effectively guards against the fearful hæmorrhage which must otberwise have taken place. It is further evident, that although this interesting arrangement is sufficient to prevent the loss of blood when the whole of it is called into action on the normal detachment of the placenta, that if a partial separation of this organ takes place in the course of gestation without delivery following, the uterus not contracting, profuse hæmorrhage must occur.

All this shows that the contraction of the uterus is the essential means employed by Nature to prevent the loss of blood on the separation of the placenta, so far as the uterine veins are concerned. The mode in which arterial hæmorrhage is guarded against, is not so well understood. The forcible contraction of the muscular uterine walls is doubtless one cause ; and probably the remarkable coils described by the uterine arteries at the place of their entrance into the placenta, by inducing an elastic retraction after laceration, may, by aiding the other known means adopted by nature in the spontaneous suppression of hæmorrhage, be another influential circumstance, especially as regards vessels of the moderate magnitude of the "curling arteries."

The exact mode in which these last-named arteries end in the commencing branches of the uterine veins implanted in the placenta, is not known ; but it cannot be doubted there is a direct continuity between the two sets of vessels. It is also certain that the arrangement and character both of the uterine and umbilical blood-vessels are such as to retard, in a remarkable degree, the velocity of the circulation ; in the former vessels, owing to the great magnitude of the veins or sinuses as they are called ; and in the latter, in consequence of the very interesting disposition of the capillaries lodged within the tufts of the chorion, and so admirably described and defined by Professor E. H. Weber, and, independently, by Mr. Dalrymple. The principal peculiarity of these umbilical capillaries is their great number, in proportion to the stems with which they are connected ; a disposition which has an influence in diminishing the rapidity of the blood's current, both by enlarging the aggregate space through which it flows in passing from the arteries into the capillaries, and also by increasing the amount of friction owing to the minute size of the individual channels. It will not be superfluous to state, that the mechanical arrangement of the uterine arteries penetrating the convex surface of the placenta, and of the umbilical arteries entering by the concave surface of that organ, both being very much coiled, especially the former, is such as must further concur in retarding the placental circulation.

The general result of all these elaborate arrangements is to allow time for that important reciprocal interchange of materials between the maternal and fœtal blood, which has already been alluded to, and on which the nourishment of the fœtus depends.

The large divisions of the uterine sinuses lodged between the lobes of the placenta communicate together, as can be proved by tracing them and also by inflation ; there is therefore a disposition which, independently of any other circumstances, would tend to cause hæmorrhage from the substance of the placenta in cases of partial separation of that organ.

We have inserted the title of Dr. Erdl's work at the head of this article, not with an intention of noticing it on the present occasion, but for the purpose of calling the attention of our readers to the fact of its publication. It consists of a numerous series of plates illustrative of the development of the chick and human embryo, with brief explanations ; and although we think that some of the drawings might have been more happily designed and executed, and that the descriptions should have been given in Latin as well as German, yet, upon the whole, it is a very useful work, and we therefore recommend it to our readers, and especially to medical societies, as a desirable addition for their libraries. It is, however, necessary to state that at present the first volume only, consisting of two parts, has been published ; the whole work is to consist of two volumes, and will, there is no doubt, be speedily completed.

The objects of Dr. Erdl's publication will be gathered from the following extract. "Under this title (Die Entwickelung des Menschen, &c.), I present to students, to teachers, and to those who are devoted to scientific pursuits, a work which is designed to supply a defect of some moment in physiological literature. My object is to give, as accurately as possible, figures of the various stages of development of the human embryo and of its individual organs and tissues, accompanied by an explanatory text. But as in the human embryo many of the earliest processes cannot be detected, I have endeavoured to supply the deficiency by means of observations made on the hen's egg."—*Prospectus.*

We shall probably, on a future occasion, have an opportunity of briefly noticing the work of Dr. Erdl, in connexion with the subjects which it more especially is designed to illustrate, the development, namely, of the body, organs, and tissues of the embryo.

